# Selective neurodegeneration generated by intravenous α‐synuclein pre‐formed fibril administration is not associated with endogenous α‐synuclein levels in the rat brain

**DOI:** 10.1111/bpa.13128

**Published:** 2022-11-02

**Authors:** Wei‐Li Kuan, Maha Alfaidi, Catherine B. Horne, Benjamin Vallin, Sarah Fox, Shaline V. Fazal, Caroline H. Williams‐Gray, Roger A. Barker

**Affiliations:** ^1^ John van Geest Centre for Brain Repair, Department of Clinical Neuroscience University of Cambridge Cambridge UK; ^2^ Department of Neurology Addenbrooke's Hospital Cambridge UK; ^3^ Wellcome Trust MRC Cambridge Stem Cell Centre Cambridge UK

**Keywords:** neurodegeneration, pre‐formed fibrils, rabies virus glycoprotein (RVG), selective vulnerability, α‐synuclein

## Abstract

Selective loss of discrete neuronal populations is a prominent feature of many neurodegenerative conditions, but the molecular basis of this is poorly understood. A central role of α‐synuclein in the selective neurodegeneration of Parkinson's disease has been speculated, as its level of expression critically determines the propensity of this protein to misfold. To investigate whether the propensity of neuronal cell loss is associated with the level of endogenous α‐synuclein expression, non‐transgenic rats were given a single intravenous administration of α‐synuclein pre‐formed fibrils (PFFs) reversibly complexed with the rabies virus glycoprotein peptide (RVG9R). The number of surviving cells in different neuronal populations was systematically quantified using unbiased stereology. Our data demonstrated that a non‐selective, transvascular delivery of α‐synuclein PFFs led to a time‐dependent loss of specific populations of midbrain (but not olfactory) dopaminergic neurons, medullary (but not pontine) cholinergic neurons, and brainstem serotonergic neurons. Contrary to the central role of endogenous α‐synuclein expression in determining the seeding and aggregation propensity of pathological α‐synuclein, we did not observe an association between the levels of α‐synuclein expression in different regions of the rodent brain (although did not ascertain this at the individual cell level) and neurodegenerative propensity. The results from our study highlight the complexity of the neurodegenerative process generated by α‐synuclein seeding. Further investigations are therefore required to elucidate the molecular basis of neurodegeneration driven by exogenous pathogenic α‐synuclein spread.

## Graphical abstract

A non‐selective, transvascular delivery of α‐synuclein pre‐formed fibrils led to a time‐dependent loss of specific populations of neurons. However, we did not observe an association between the levels of α‐synuclein expression in different regions of the rodent brain and neurodegenerative propensity. Results from our study highlight the complexity of the neurodegenerative process generated by α‐synuclein seeding.

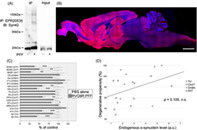

## 1 INTRODUCTION

The presence of α‐synuclein pathology, together with the selective degeneration of discrete cell populations, including dopaminergic neurons in the substantia nigra, cholinergic neurons in the dorsal motor nucleus, as well as serotonergic neurons in the raphe nucleus, are prominent pathological features defining Parkinson's disease (PD) [1]. Recent studies have suggested a positive association between the severity of α‐synuclein pathology and endogenous α‐synuclein expression [2, 3]. However, the association between α‐synuclein expression levels and neuronal death remains unclear, with several reports even demonstrating selective neurodegeneration in brain areas in the absence of α‐synuclein pathology in PD (e.g., median raphe nucleus [4] and pre‐supplementary motor cortex [5]).

Previously, direct stereotaxic injections of α‐synuclein pre‐formed fibrils (PFFs) into the brain have been shown to seed α‐synuclein pathology and neurodegeneration in nontransgenic animals [6, 7]. However, the pattern of cell loss generated in these studies is critically dependent on the site of injection, which compromises the utility of such models to study selective neurodegeneration. To overcome this, we recently developed a novel approach using a modified rabies virus glycoprotein (RVG9R), which enables a transvascular, non‐selective delivery of PFFs through systemic administration [8]. RVG9R interacts specifically with the nicotinic acetylcholine receptor on neuronal cells for cargo delivery [9, 10]. Unlike the systemic delivery of α‐synuclein PFF, which is unable to generate central nervous system (CNS) neurodegeneration [11], we have been able to demonstrate selective neuropathology that resembles some aspects of the pathology seen in patients with early PD using an RVG‐mediated PFF delivery approach. We now seek to quantify the long‐term pattern of cell loss using our novel model, to investigate the association between neurodegenerative propensity and physiological α‐synuclein expression within defined regions of the CNS.

## 2 MATERIALS AND METHODS

### 2.1 Ethics

All animal experiments were done in accordance with a project licence held under the Animals (Scientific Procedures) Act 1986, Amendment Regulations 2012, following ethical review by the University of Cambridge animal welfare and ethical review body.

### 2.2 Preparation of α‐synuclein PFFs

α‐synuclein PFFs were prepared using the protocol published previously [12]. In short, full‐length human recombinant α‐synuclein monomers (rPeptide) were resuspended in 10 mM Tris–HCl, 50 mM NaCl, pH 7.6 at a concentration of 5 μg/μl. They were then placed on a 37°C thermomixer, agitated for 7 days at 1000 revolutions per minute (rpm), and stored in a −80°C freezer. Immediately before use, the PFFs were thawed and diluted to 0.1 μg/μl in phosphate‐buffered saline (PBS) and sonicated for 60 min with an ultrasonic waterbath (Decon FS100b) (Figure S1).

### 2.3 Transmission electron microscopy

α‐Synuclein PFF samples, before and after sonication, were adsorbed onto glow discharged (GlowCube, Quorum Technologies, Laughton, UK) 400 mesh, copper, carbon film grids (EM Resolutions, Saffron Walden, UK) for 30 s, rinsed twice with deionised water and negatively stained with 1.5% aqueous uranyl acetate for 30 s. They were blotted on filter paper and viewed in a FEI Tecnai G2 (FEI Ltd, Oregon, USA operated at 20 kV). Images were recorded with an AMT XR60B camera running Deben software (AMT, Woburn, USA).

### 2.4 Peptide/PFFs complex formation

Peptide RVG9R (YTIWMPENPRPGTPCDIFTNSRGKRASNGGGGRRRRRRRRR), composed of a short domain originating from the rabies virus glycoprotein and nine C‐terminally conjugated d‐arginines [9, 10], was synthesized by ProteoGenix SAS (France). To prepare the complex for administration, RVG9R peptides and α‐synuclein PFFs were mixed in PBS in a glass vial (Supelco) and left on an orbital shaker (Stuart SSM1, 60 rpm), for 30 min at room temperature before use. The molecular weight of the sonicated α‐synuclein PFFs was assumed to be 14.5 kDa, and the peptide/protein complex was mixed at a 10:1 molar ratio.

### 2.5 Animal procedures

Female Sprague–Dawley rats were housed with unrestricted access to food and water unless otherwise stated. Animals were housed in groups of four on a 12 h light/dark cycle. Consistent with our previous report [8], a total volume of 300 μl of the peptide/protein complex, containing 100 μg of the PFFs, was administered via the lateral tail vein at 16 weeks of age. All animals were behaviourally assessed every 3 months and sacrificed at 6, 12, and 18 months post‐injection. Information on the number of animals used in this study is provided in Table S1.

### 2.6 Behavioural assessments

#### 2.6.1 Staircase test

Two food pellets (Kellogg's coco pops) were placed bilaterally on the staircase at each of the seven graded steps, which represented different levels of reaching difficulty. The rats were fasted overnight before each testing session, and the number of pellets eaten by the animals was recorded. A stable baseline was reached by testing for five consecutive days pre‐lesion, with the post‐lesion results expressed as a percentage of change from the baseline [13].

#### 2.6.2 Rotarod test

Rotarod test was performed using an accelerating rotarod for two large rats (Panlab), with the speed of rotation increased by 1 rpm every 30 s until it reached 40 rpm. One day prior to testing, all animals were habituated to the experimental room and the rotarod. On the test day, rats were individually placed on the rod, and the latency and speed at which an individual rat fell off the rotarod was recorded [14].

#### 2.6.3 Buried food test

Animals were familiarised with the food stimuli (Kellogg's coco pops) and fasted overnight. A food pellet was buried in the middle of the home cage and the latency to retrieve the pellet was recorded. If the animal failed to find the buried food after 15 min, the test was stopped with 900 sec recorded as its latency score [8].

#### 2.6.4 Cylinder test

The rats were put individually in a glass cylinder (21 cm diameter, 45 cm height) for 5 min, and the number of supporting contacts (full appositions of the paws with open digits to the cylinder walls) performed independently with the left and the right forepaw were counted. The number of rearing events was also recorded [15].

#### 2.6.5 Novel object recognition

The animals were left in an open‐field with two identical objects for 10 min of free exploration. After a 60 min delay they were put back into the open‐field for another 10 min with one of the original objects replaced with a new one. The discrimination ratio [**Time**_(novel object)_−**Time**_(familiar object)_]/[**Time**_(novel object)_+**Time**_(familiar object)_], where a positive score indicated that the animal spent more time with the novel object, as well as the preference index [**Time**_(novel object)_]/[(**Time**_(novel object)_+**Time**_(familiar object)_] × 100%), where the ratio indicates the percentage of time spent exploring the novel object, were both calculated [16].

#### 2.6.6 Marble burying test

The clean cages were filled with 4.5 cm corncob bedding, followed by overlaying 20 black glass marbles (15 mm diameter) equidistant in a 4 × 5 arrangement. Animals were placed individually in the cage and after a 30 min exploration period, the numbers of fully buried marbles and unburied marbles were recorded [8].

### 2.7 Immunohistochemistry

After perfusion of the rats at 6‐, 12‐, and 18‐months post‐lesion, the brains were quickly removed, post‐fixed overnight, and transferred to 30% sucrose until they sank. Sections were cut at 35 μm in either the coronal or sagittal plane, and a 1:6 series of sections was immunostained. After blocking with 0.3% Triton X‐100 with 5% normal donkey serum for 1 h, coverslips or sections were incubated overnight with various primary antibodies including tyrosine hydroxylase (1:1000, Millipore MAB318), choline acetyltransferase (1:250, Millipore AB144P), glutamate decarboxylase‐67 (1:1000, MAB5406), tryptophan hydroxylase (1:1000, Sigma T0678), or rodent‐specific α‐synuclein (1:200, CST D37A6). The sections were visualised either by immunofluorescence (Alexa 568 and Alexa 647, 1:1000, Molecular Probes) or by diaminobenzidine. Staining was also done in all experiments without the primary antibody which served as a negative control in all cases.

### 2.8 Western blot

Cellular proteins from the brain or peripheral tissues were extracted using radioimmunoprecipitation assay buffer, supplemented with complete protease inhibitor. Total protein concentration was determined by the bicinchoninic acid (BCA) assay. A total amount of 10–30 μg of protein per lane was loaded onto a 10% pre‐cast gel (ThermoFisher) for sodium dodecylsulphate‐polyacrylamide gel electrophoresis and electroblotted onto 20 nm polyvinylidene difluoride membranes (GE). Membranes were then blocked in TBS Tween buffer (Pierce) supplemented with 5% dry skimmed milk, and incubated with antibodies against PSD95 (1:1000, Abcam Ab18258), synaptophysin (1:1000, Abcam Ab8049), or β‐actin (1:5000, Santa Cruz sc‐47778) at 4°C overnight. The membranes were then rinsed and incubated with horseradish‐peroxidase‐conjugated secondary antibody (1:5000, Santa Cruz) for 1 h at room temperature. Membranes were developed with the SuperSignal West Pico chemiluminescence method (Life Technologies).

### 2.9 Immunoprecipitation

Flash frozen cortex was homogenised in 1% Triton‐X100 with Complete protease inhibitors. Protein concentration was quantified using a BCA assay. Homogenate containing 1 mg protein was ultracentrifuged at 120,000 *g* (Beckman Optima Max‐XP) for 1 h at 4°C. The pellet was dispersed in 1% Triton‐X100 using a needle to produce a homogenate containing a Triton‐insoluble fraction. From this homogenate, a 5% input was removed for subsequent immunoblotting. The remaining triton‐insoluble fraction was incubated overnight at 4°C with the IP antibody against α‐synuclein (1:100, Abcam ab212184). The mixture was then incubated with 25 μl Protein G Dynabeads according to the manufacturer's instruction. The protein of interest was eluted using NuPAGE LDS sample buffer and separated by electrophoresis on a 4%–12% polyacrylamide gel. Immunoblotting was performed using 5% milk blocking with overnight incubation at 4°C with an α‐synuclein antibody (1:1000, BD 610787). The membrane was incubated with a horseradish‐peroxidase‐conjugated anti‐mouse antibody (1:20,000, Sigma A9917) for 1 h, and visualised with chemiluminescence.

### 2.10 Microscopy and stereology

Imaging was performed using the BX50 microscope (Olympus). The total number of surviving neurons in various brain regions was estimated by unbiased stereology using a combination of the Cavalieri probe and optical fractionator, with the Stereo Investigator 9.10.3 software (MBF Bioscience). The average final section thickness was 27 μm. The estimated coefficient of error of individual neuronal populations is provided in Table S2.

### 2.11 Microscopy and CellProfiler analysis

Fluorescent imaging was performed using the wild‐field DMi8 moving stage microscope (Leica), or TCS SP2 confocal microscope (Leica). Stitched images of the entire tissue section were generated with the 4× objective. The same imaging parameter (gain/exposure) was used to capture fluorescent images of endogenous α‐synuclein expression across different neuronal populations. A CellProfiler (version 4.0.7) pipeline was designed to quantify the expression of rodent‐specific α‐synuclein among different neuronal subsets. In short, the “ColorToGray” module was used to convert the Leica image into a grayscale image for individual channels. The images were then filtered with the “GaussianFilter” module for noise reduction. The contour of individual neurons was identified using their respective markers and defined with the “IdentifyPrimaryObjects” module. The “RelateObjects” module was used to identify rodent‐specific α‐synuclein colocalised within the cell bodies, with the “MeasureObjectIntensity” module applied to quantify the intensity of rodent‐specific α‐synuclein expression. α‐Synuclein fluorescence intensity was normalised by the number of neurons per field‐of‐view. Because of the high heterogeneity of endogenous α‐synuclein expression even within the same neuroanatomical region [3], an average of 50–100 neurons were analysed and averaged per region per animal, in order to better reflect the level of endogenous α‐synuclein expression within particular neuroanatomical regions.

### 2.12 Statistical analysis

All behavioural data were analysed by Generalized Estimating Equations, thus taking into account the different sample sizes over time. All other quantitative data were analysed using nonparametric methods including Kruskal–Wallis test (performed at all nine levels of each time‐point × group, followed by multiple comparison adjustments), and Spearman's correlation. Cross‐validation analysis with a repeated random sub‐sampling approach, as well as binominal analysis, were all performed as described by us previously [17]. All analyses were performed using SPSS Release 27.0.0. Data are presented either in line graph showing group mean and standard error of the mean, or in box plots showing group median and 1.5 interquartile range. No statistical methods were used to predetermine sample sizes, but our sample sizes are similar to those reported in previous publications [6, 7, 8]. Group samples were not randomised; data collection, but not data analysis, was performed blind to the conditions of the experiments. No collected data were excluded from analysis.

## 3 RESULTS

**FIGURE 1:**
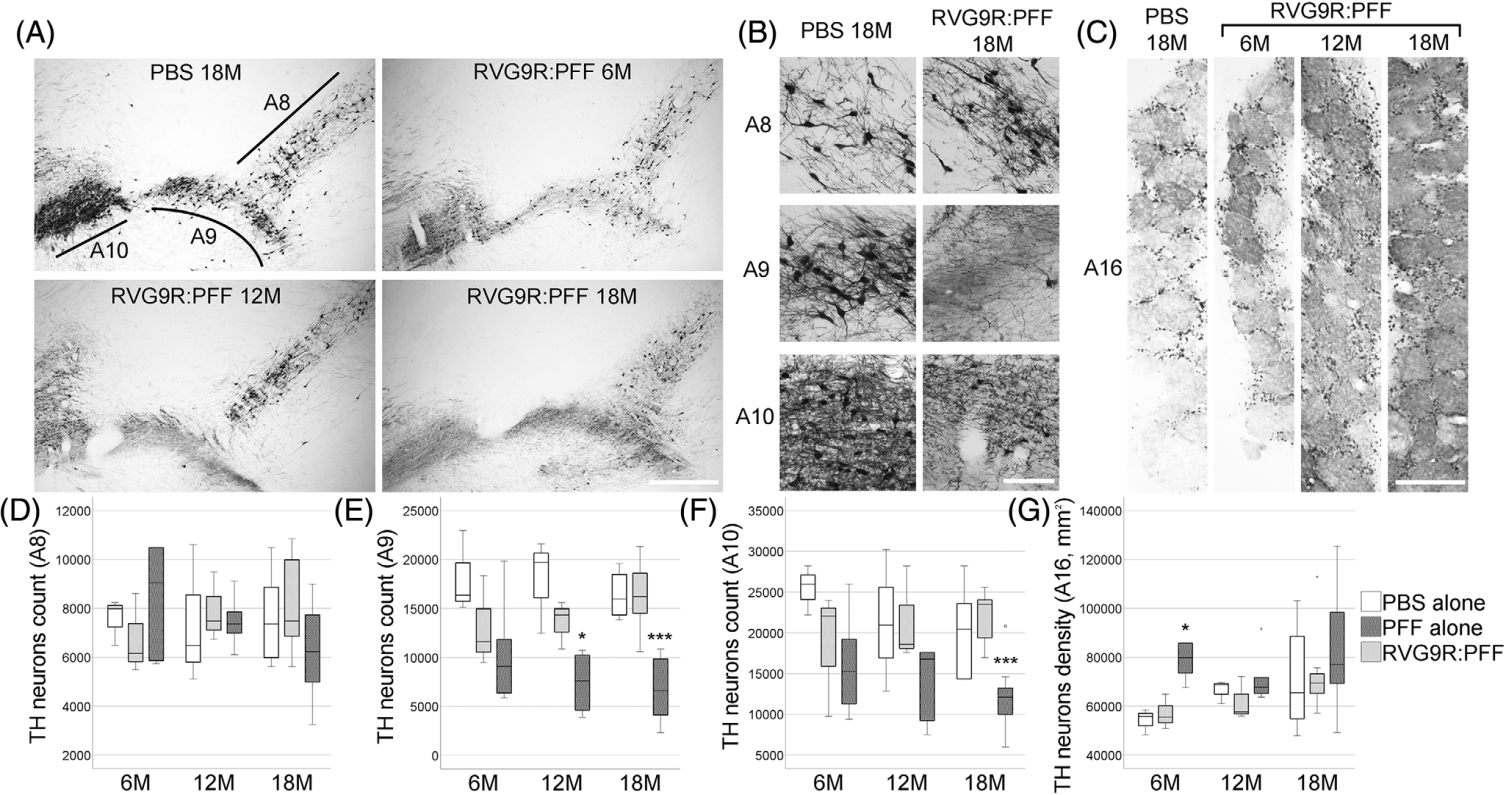
(A) Representative pictures and (B) higher magnification images for discrete neuronal populations, to demonstrate the survival of tyrosine hydroxylase (TH)‐positive dopaminergic neurons in the retrorubral field (A8), substantia nigra pars compacta (A9), and ventral tegmental area (A10). (C) Representative images of the olfactory bulb dopaminergic neurons (A16). (D) Quantification of neuronal survival at different stages post‐lesion. *p < 0.05, ***p < 0.001 compared with PBS group at the same time point. Data were analysed using the Kruskal–Wallis test adjusted for multiple comparisons. *n* = 4, 8, and 10–12 per group at 6, 12, and 18 months post‐injection, respectively. Scale bars, 500 μm in (A, C), 100 μm in (B).

We studied changes in behavioural performance in different functional domains of non‐transgenic, Sprague–Dawley rats up to 18‐months after a single, intravenous administration of RVG9R:PFF, PFF alone, or PBS control. We did not include animals receiving RVG9R alone, as we (and others) have previously shown that the RVG peptide when administered on its own does not generate any CNS pathology [10, 18, 19]. There was a significant deterioration in forelimb control in the RVG9R:PFF rats over time (*p* = 0.049, Figure S2A), although gross motor coordination and spontaneous forelimb use was not affected (Figure S2B–D). Consistent with the motor impairment, there was a significant decrease in the number of surviving A9 (χ^2^ (2) = 29.913, *p* < 0.001) and A10 dopaminergic neurons (χ^2^ (2)= 20.319, *p* < 0.001), supporting the cytotoxicity of pathogenic α‐synuclein in both the nigrostriatal and mesolimbic systems [20]. This was in line with our previous data [8], where we confirmed the loss of midbrain dopaminergic neurons, rather than simply a downregulation of tyrosine hydroxylase expression, by quantifying the number of vesicular monoamine transporter neurons in this region. This moderate level of midbrain dopaminergic neurodegeneration also explains the lack of a strong behavioural phenotype, which is normally only observed when the loss of A9 dopaminergic neurons is greater than 70% [21]. Intriguingly, dopaminergic neurons in the A8 (retrorubral field), which are morphologically indistinguishable from the A9 and A10 neurons, and are anatomically connected to the substantia nigra and ventral tegmental area [22], showed no α‐synuclein toxicity (Figure 1). There was also an increase in the A16 dopaminergic neurons in the olfactory bulb (χ^2^ (2) = 9.280, *p* = 0.01; Figure 1), consistent with our previous findings [8].

**FIGURE 2:**
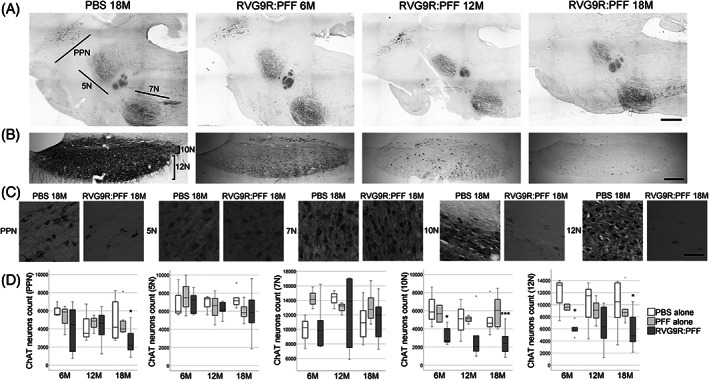
Representative tile‐scan pictures to demonstrate the survival of choline acetyltransferase (ChAT)‐positive cholinergic neurons in the (A) pedunculopontine nucleus (PPN), trigeminal nucleus (5N), facial nucleus (7N), (B) dorsal motor nucleus (10N), and hypoglossal nucleus (12N). (C) Representative images of individual neuronal populations, and (D) quantification at different stages post‐lesion. *p < 0.05, ***p < 0.001 compared with the PBS group at the same time point. Data were analysed using the Kruskal–Wallis test adjusted for multiple comparisons. *n* = 4, 8, and 10–12 per group at 6, 12, and 18 months post‐injection, respectively. Scale bars, 1 mm in (A), 500 μm in (B), 100 μm in (C).

**FIGURE 3:**
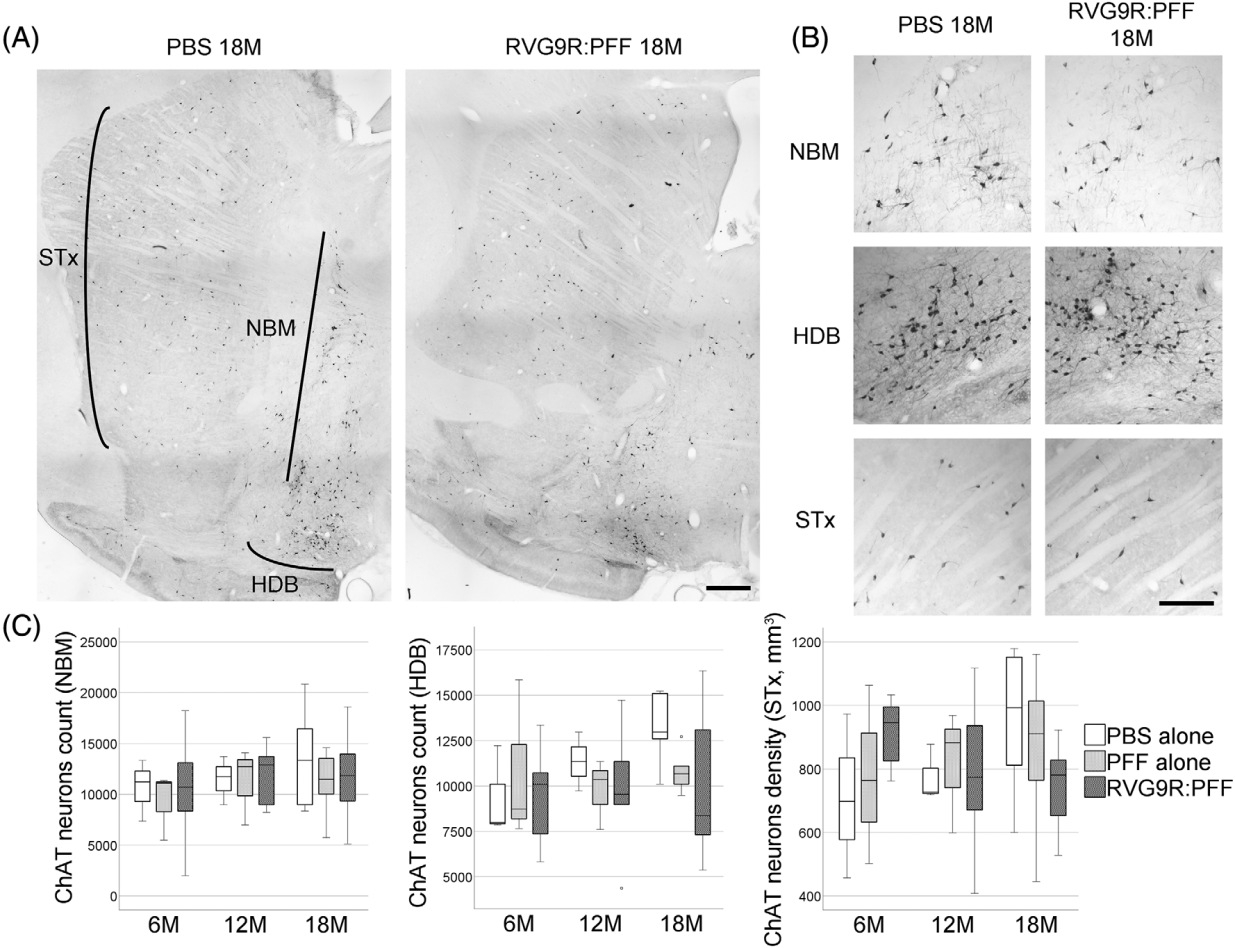
(A) Representative tile‐scan pictures to demonstrate the survival of choline acetyltransferase (ChAT)‐positive cholinergic neurons in the striatum (STx), Nucleus Basalis of Meynert (NBM), and horizontal diagonal band of Broca (HDB). (B) Representative images of individual neuronal populations, and (C) quantification at different stages post‐lesion. Data were analysed using the Kruskal–Wallis test adjusted for multiple comparisons. *n* = 4, 8, and 10–12 per group at 6, 12, and 18 months post‐injection, respectively. Scale bars, 1 mm.

**FIGURE 4:**
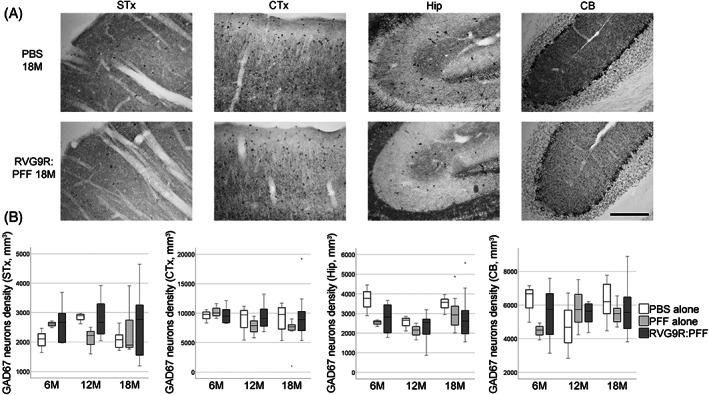
(A) Representative pictures, and (B) quantification to demonstrate the survival of glutamate decarboxylase‐67 (GAD67)‐positive GABAergic neurons in the striatum (STx), somatosensory cortex (CTx), hippocampus (Hip), and cerebellum (CB), at different stages post‐lesion. Data were analysed using the Kruskal–Wallis test adjusted for multiple comparisons. *n* = 4, 8, and 10–12 per group at 6, 12, and 18 months post‐injection, respectively. Scale bars, 500 μm.

Given that we are modelling PD, we also sought to look for other relevant pathology outside the dopaminergic system. We found that there was a significant reduction in several populations of brainstem cholinergic neurons, including those of the pedunculopontine nucleus (χ^2^ (2) = 6.638, *p* = 0.036), dorsal motor nucleus (χ^2^ (2) = 21.720, *p* < 0.001), and hypoglossal nucleus (χ^2^ (2) = 12.791, *p* = 0.002), but not the trigeminal or facial nuclei (Figure 2). A trend for hypoglossal cholinergic neurodegeneration (*p* = 0.073) has recently been reported in patients with incidental LB disease, but intriguingly not in idiopathic PD patients [23]. On the other hand, none of the forebrain cholinergic neurons (Figure 3) were found to be susceptible to α‐synuclein toxicity in our model. This was in contrast to a previous report, showing the susceptibility of striatal and cortical cholinergic neurons to viral vector‐mediated overexpression of wild‐type α‐synuclein [24]. Furthermore, none of the GABAergic groups assessed were significantly affected by RVG9R:PFF injections (Figure 4), in line with previous studies reporting that GABAergic neurons are more resistant to develop pathology after PFF challenges [3].

**FIGURE 5:**
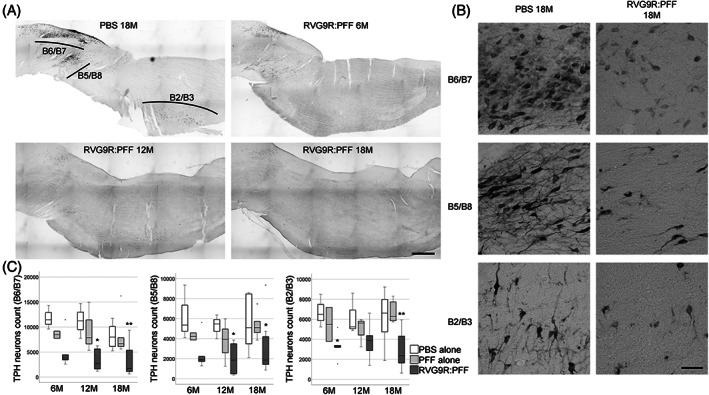
(A) Representative tile‐scan pictures and, (B) higher magnification images for individual neuronal subsets, to demonstrate the survival of tryptophan hydroxylase (TPH)‐positive serotonergic neurons in the dorsal raphe nucleus (B6/B7), pontine raphe nucleus (B5/B8), and the raphes obscurus/magnus (B2/B3). (C) Quantification of individual neuronal populations at different times post‐injection of the RVG9R:PFF. *p < 0.05, **p < 0.01 compared with PBS group at the same time point. Data were analysed using the Kruskal–Wallis test adjusted for multiple comparisons. *n* = 4, 8, and 10–12 per group at 6, 12, and 18 months post‐injection, respectively. Scale bars, 1 mm in (a), 100 μm in (B).

Loss of serotonergic neurons in the medulla is prominent in patients dying with an α‐synucleinopathy (including both PD and multiple system atrophy [4, 25]), but to our knowledge this has never been studied in the context of a PFF challenge. All serotonergic populations were assessed in our model, including the dorsal (B6/B7, χ^2^ (2) = 23.911, *p* < 0.001) and pontine (B5/B8, χ^2^ (2) = 16.920, *p* < 0.001) raphe nucleus, as well as the raphe obscurus/magnus (B2/B3, χ^2^ (2) = 21.247, *p* < 0.001; Figure 5), and all were found to have been significantly affected by the RVG9R:PFF injection.

To investigate whether the specific pattern of neurodegeneration in the RVG9R:PFF rats could be detected at a regional level, we sought to compare the level of synaptic protein expression between treatments. There were, however, no significant group differences in the expression of PSD95 (postsynaptic marker) and synaptophysin (presynaptic marker) in the midbrain and brainstem samples (Figure S3), supporting the restricted specificity of α‐synuclein toxicity to only particular neuronal populations.

**FIGURE 6:**
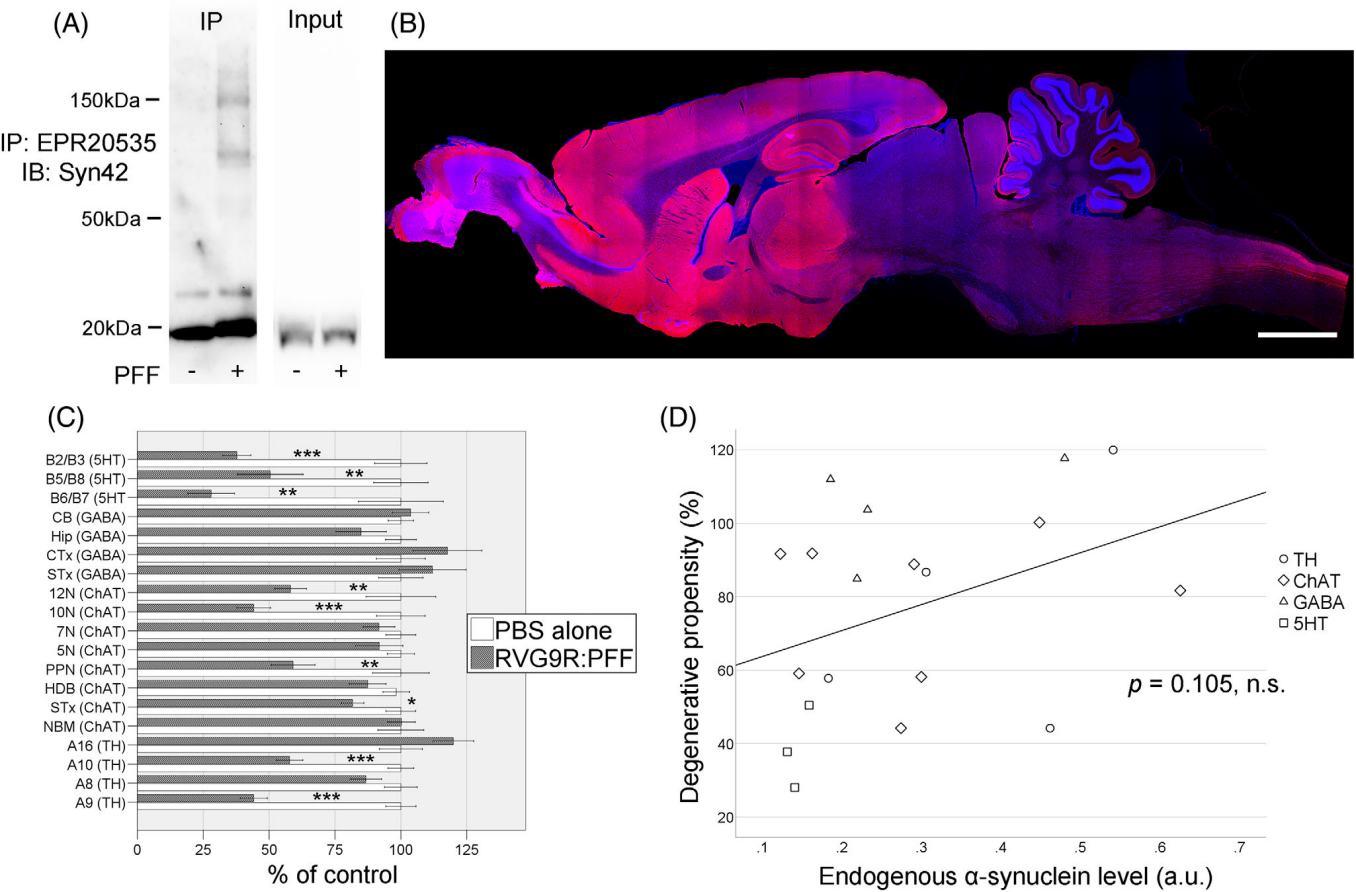
(A) Aggregated α‐synuclein is detectable using immunoprecipitation (IP) in the cortex of RVG9R:PFF animals. (B) Representative image of physiological α‐synuclein expression (red) in a control animal using the rodent‐specific α‐synuclein antibody. (C) the degenerative propensity of discrete neuronal populations to α‐synuclein toxicity, showing (D) no association with endogenous α‐synuclein expression. *p < 0.05, **p < 0.01, ***p < 0.001 compared with PBS group. Data were analysed using the Kruskal–Wallis test adjusted for multiple comparisons. *n* = 10–12 per group. Nuclei were counterstained with Hoechst (blue). Scale bars, 4 mm in (B).

Unlike the intracerebral PFF seeding models that leads to profound, Lewy body‐like aggregates [6, 7], α‐synuclein pathology generated by RVG9R‐mediated PFF delivery was diffuse in nature and thus difficult to systematically quantify [8]. We therefore performed immunoprecipitation using pan‐α‐synuclein antibodies to confirm the presence of Triton‐insoluble, high molecular weight α‐synuclein aggregation only in the lesioned animals (Figure 6A). Even though we used a transvascular delivery of α‐synuclein PFFs that entered the whole brain, our model is unique as it enables us to quantify cell loss in discrete neuronal populations. By evaluating the level of physiological α‐synuclein expression in control animals using a rodent‐specific α‐synuclein antibody (Figure S4) with the pattern of selective neurodegeneration in the lesioned animals, we were able to estimate the neurodegenerative propensity of discrete neuronal populations (although not at the level of individual neurons) at 18‐months post‐lesion (surviving neurons in $\left(\mathrm{RVG}9R:\mathrm{PFF}\right)/\left(\mathrm{PBS}\;\text{alone}\right)$; Figure 6B,C). There was no significant association between the level of endogenous expression of α‐synuclein and the severity of PFF‐induced neurodegeneration at the level of individual brain nuclei (Figure 6D, *p* = 0.105). We then attempted to cross‐validate our approach by randomly selecting 12 out of the 19 brain regions and repeated the process 40 times, to verify if our results could be attributed to random sampling error. In line with our initial finding, 36 out of the 40 tests showed no significant association between neurodegenerative propensity and the level of endogenous α‐synuclein expression (*p* = 0.138, *q* = 0.05). Our result also holds when results from brain regions with potentially saturating levels of endogenous α‐synuclein expression were excluded (cortex, striatum, hippocampus, *p* = 0.114). Our data therefore suggests that the expression of endogenous α‐synuclein in discrete populations of neurons does not underlie the selective vulnerability of the neuronal loss seen in this systemic RVG9R:PFF model of α‐synucleinopathy.

## 4 DISCUSSION

Although the seeding and aggregation propensity of α‐synuclein is governed by the levels of α‐synuclein expression and the anatomical circuitry, our data is the first to address the relationship between physiological α‐synuclein expression in neuronal populations and their neurodegeneration propensity. Our results highlight the complexity of the neurodegenerative process triggered by misfolded α‐synuclein, and that the heterogeneity of cell loss, cannot simply be attributed to the level endogenous neuronal α‐synuclein expression in a particular brain region.

Because of its central role in the pathogenesis of PD and related disorders, α‐synuclein is often considered to be a major therapeutic target. Duplication and triplication of the *SNCA* gene, which encodes the α‐synuclein protein, causes familial forms of PD [26, 27]. In line with this, the overexpression of α‐synuclein in neurons results in neuronal loss [28, 29], while animals without endogenous α‐synuclein expression are resistant to PFF toxicity [6, 12]. However, our data indicates that a threshold level of physiological α‐synuclein expression appears to be sufficient for pathology to develop. Consistent with this, post‐mortem human brain analysis demonstrates a comparable level of α‐synuclein expression between A9 and A10 dopaminergic neurons [30], despite the fact that the A9 neurons are more affected and lost in PD. Thus other factors have been linked to this selective vulnerability, including the presence or not of intrinsic pace‐making activity as well the level of L‐type calcium channels [31, 32].

Other risk factors driving the selective vulnerability of neurons to α‐synuclein toxicity have also been proposed. Midbrain dopaminergic neurons, medullary cholinergic neurons, and raphe serotonergic neurons all share similar characteristics by having extensive networks of long, unmyelinated axons. It is likely that the proteostatic burden and bioenergetic demand associated with such a phenotype is substantial. Indeed, it has been recently demonstrated that increasing the complexity of axonal arborisation through genetic manipulation is associated with an increased susceptibility to neurotoxic insult [33]. On the other hand, as non‐neuronal populations, such as astrocytes and microglia, also actively participate in handling misfolded α‐synuclein, regional heterogeneity of the astrocytic and microglial population may also contribute to the selective neurodegeneration observed in our model [34, 35].

Our current result from 19 different neuroanatomical regions covering four different neurotransmitter systems, indicates that further research is still required to elucidate the molecular basis of neurodegeneration driven by pathogenic α‐synuclein, as well as the potential interplay between α‐synuclein and other neurodegenerative proteins in driving PD‐related deficits. Several recent studies have demonstrated little to no association between pathological α‐synuclein aggregation and neuronal loss in experimental models of synucleinopathy [36, 37]. Analysis of these studies are nonetheless restricted to the nigrostriatal dopaminergic system, which does not really reflect the complex pattern of neurodegeneration observed in sporadic PD. Indeed, the relationship between α‐synuclein pathology and cell loss remains unclear, with neither the phosphorylation nor fibrillisation of this protein appearing to drive neurodegeneration [38, 39, 40]. Furthermore, recent current clinical trials targeting misfolded α‐synuclein have not shown clear clinical benefits [41, 42], again suggesting that α‐synuclein might not solely explain the cell loss seen in PD. In agreement with this is the fact that the neuroprotective potential of a glucagon‐like peptide‐1 (GLP‐1) receptor agonist, which significantly increased pathological α‐synuclein load in an animal model of α‐synucleinopathy [43], has recently been shown to be a possible disease‐modifying treatment in PD patients [44].

There are, however, important limitations in our study. Firstly, an important role of physiological α‐synuclein is to regulate synaptic function, with several reports demonstrating that the pathological transformation of this protein takes place in the presynaptic nerve terminal [45, 46]. It is therefore possible that the neurodegenerative process is governed by the level of presynaptic, rather than somatic, α‐synuclein expression. Because of the extensive area that A9 dopaminergic neurons innervate, for example, this hypothesis is very difficult to validate in vivo. In relation to this, it has been demonstrated in recent studies that direct injections of α‐synuclein PFFs to the soma of nigral dopaminergic neurons (A9) and pontine cholinergic neurons (pedunculopontine nucleus) are also able seed and generate significant cell loss [47, 48], supporting in part our suggested hypothesis. Secondly, as we were unable to unbiasedly quantify the degree α‐synuclein toxicity in different neuronal populations, because of the diffuse nature of pathology generated, any interaction between α‐synuclein pathology and cell loss at the individual cell level could not be explored with the analytical tools we used. Thirdly, although the RVG9R peptide mediates cargo delivery to the brain without apparent regional selectivity [8, 49], coupled to the fact that there are no substantial regional differences in the expression of the nicotinic acetylcholine receptor (relevant to RVG9R delivery) [50], differences in transduction efficiency between individual neuronal subtypes is still possible, which could have led to the results we now report. Finally, PFF‐induced seeding is known to be dependent on the presence of a threshold level of endogenous α‐synuclein. It would therefore be of interest in the future to see whether there is any neurodegeneration in α‐synuclein knockout rats (which are now commercially available) treated with the transvascular RVG9R:PFF complex.

## AUTHOR CONTRIBUTIONS

Wei‐Li Kuan conceived the experiments. Wei‐Li Kuan, Maha Alfaidi, Catherine B. Horne, Benjamin Vallin, Sarah Fox, and Shaline V. Fazal designed and performed experiments; collected, analysed, and interpreted data. Wei‐Li Kuan and Roger A. Barker wrote the manuscript. Roger A. Barker and Caroline H. Williams‐Gray supervised experiments. All authors had final approval of the submitted manuscript.

## CONFLICT OF INTEREST

The authors declare that they have no conflict of interest.

## Supporting information


**Appendix S1:** Supporting Information

## Data Availability

Data sharing is not applicable to this article as no new data were created or analyzed in this study.
