# Supplementary material for: Selective neurodegeneration generated by intravenous α-synuclein pre-formed fibril administration is not associated with endogenous α-synuclein levels in the rat brain
Source: Brain Pathol. Author manuscript; Available in PMC 2023 May 4. (PMC10154377; doi:10.1111/bpa.13128)
Supplement: Appendix S1: Supporting Information [file EMS170593-supplement-Appendix_S1__Supporting_Information.docx]

**Selective neurodegeneration generated by intravenous α-synuclein pre-formed fibril administration is not associated with endogenous α-synuclein levels in the rat brain**

Running title: α-Syn expression and neurodegeneration

Wei-Li Kuan^1*^, Maha Alfaidi^1^, Catherine B. Horne^1^, Benjamin Vallin^1^, Sarah Fox^1^, Shaline V. Fazal^1^, Caroline H. Williams-Gray^1^, and Roger A. Barker^1,2,3^

^1^John van Geest Centre for Brain Repair, Department of Clinical Neuroscience, University of Cambridge, Cambridge CB2 0PY, U.K. ^2^Department of Neurology, Addenbrooke's Hospital, Cambridge CB2 0QQ, U.K. ^3^Wellcome Trust- MRC Cambridge Stem Cell Centre, Cambridge CB2 1QR, U.K.

*To whom correspondence should be addressed, email: [wlk21@cam.ac.uk](mailto:wlk21@cam.ac.uk)

The authors have declared that no conflict of interest exists


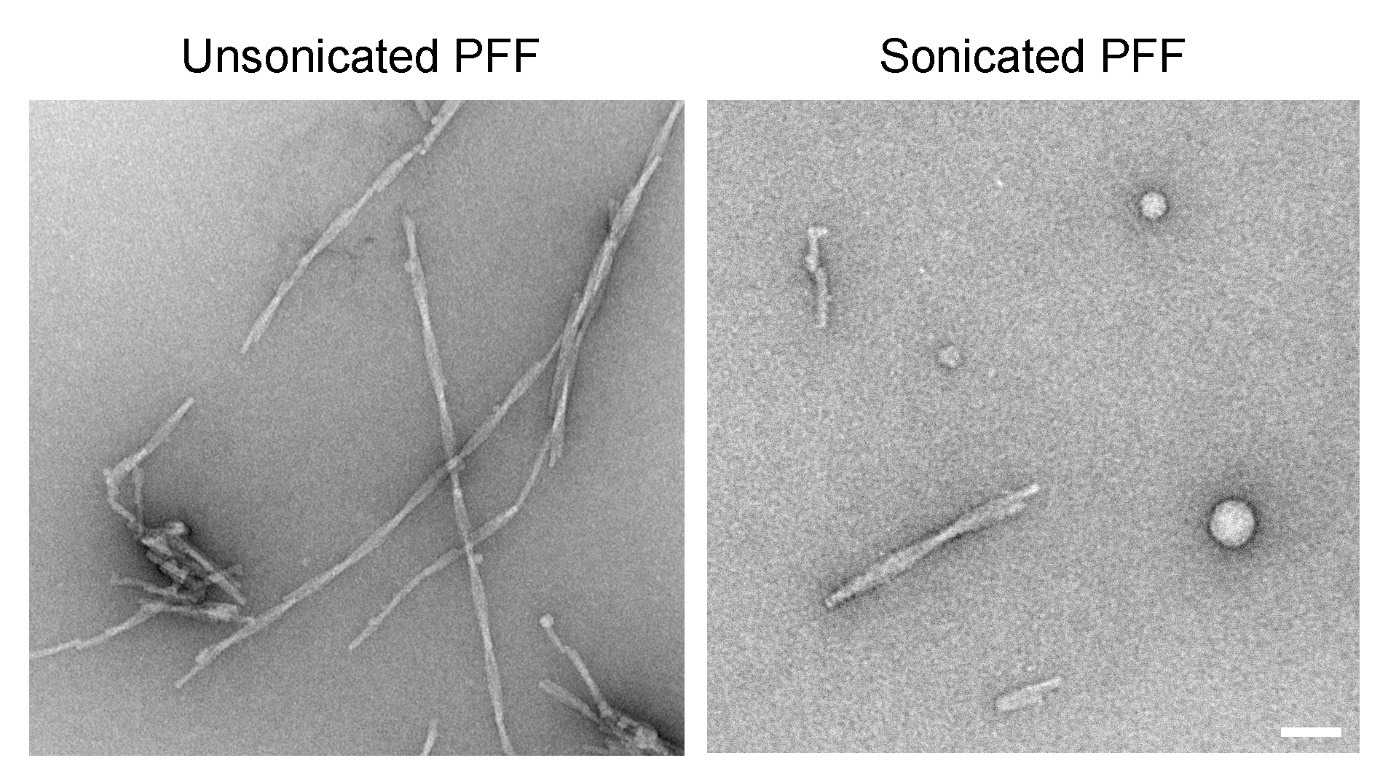


**Supplementary Figure 1.** Representative TEM images showing the ultrastructure of α-synuclein PFFs, before and after sonication. Scale bars, 100nm.


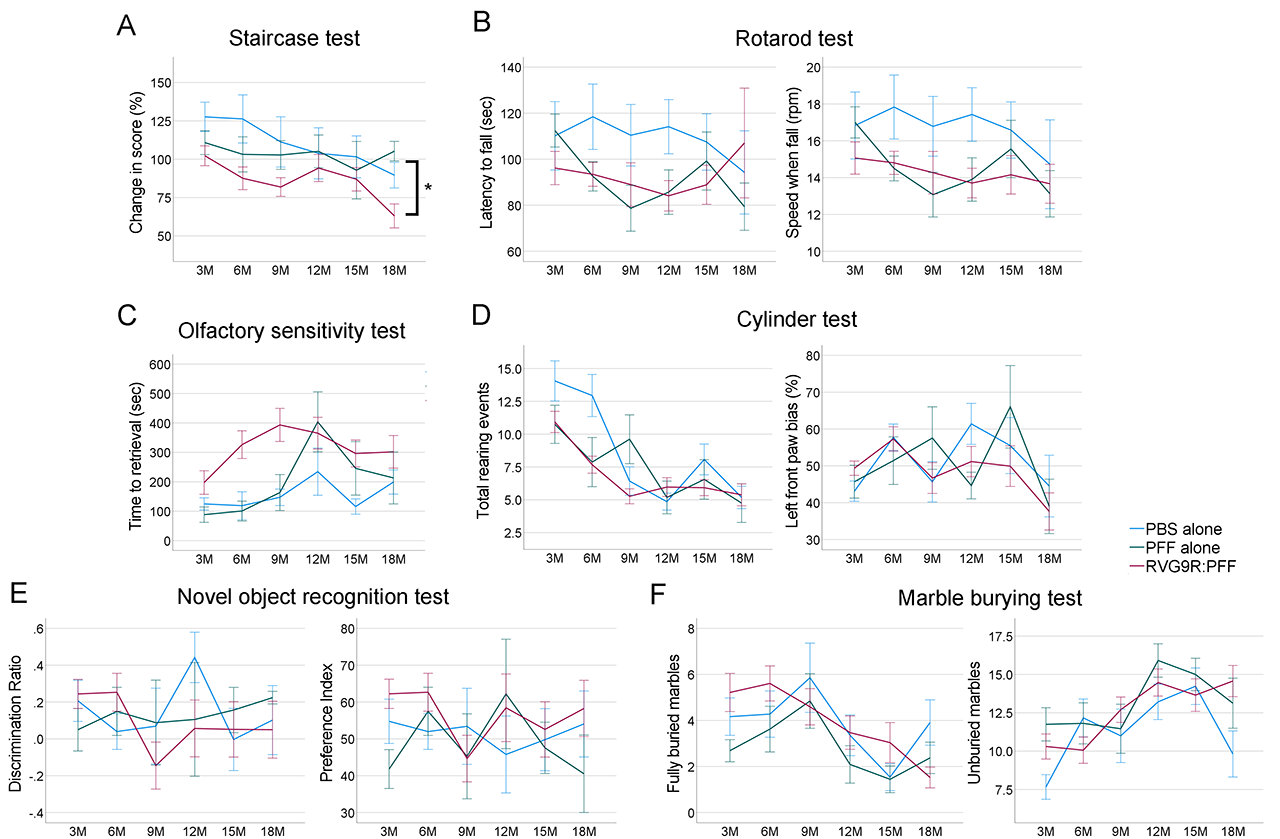


**Supplementary Figure 2.** Longitudinal changes in behaviour. (**A**) Animals in receipt of a single peripherally delivered injection of RVG9R:PFF performed significantly worse over time in the staircase test, which evaluates fine motor control of the forelimb (β=25.876, Wald χ^2^=7.618, *p*=0.006 vs. PBS alone group; β=16.453 Wald χ^2^=3.877, *p*=0.049 vs. PFF alone group). (**B**) No significant group difference was found in the rotarod test, which evaluates gross motor control. (**C**) There was a significant time effect (β=40.343, Wald χ^2^=4.298, *p*=0.038), but no group difference in the buried food test, which evaluates olfactory sensitivity. (**D**) No significant group difference was found in the cylinder test, which evaluates spontaneous forelimb use. (**E**) No significant group differences were found in the sensitivity of recognition memory using novel object recognition test. (**F**) There was no group difference in compulsive-like behaviours using marble-burying test. Results were obtained at 3-18 months post-injection, and was analysed using Generalized Estimating Equations.


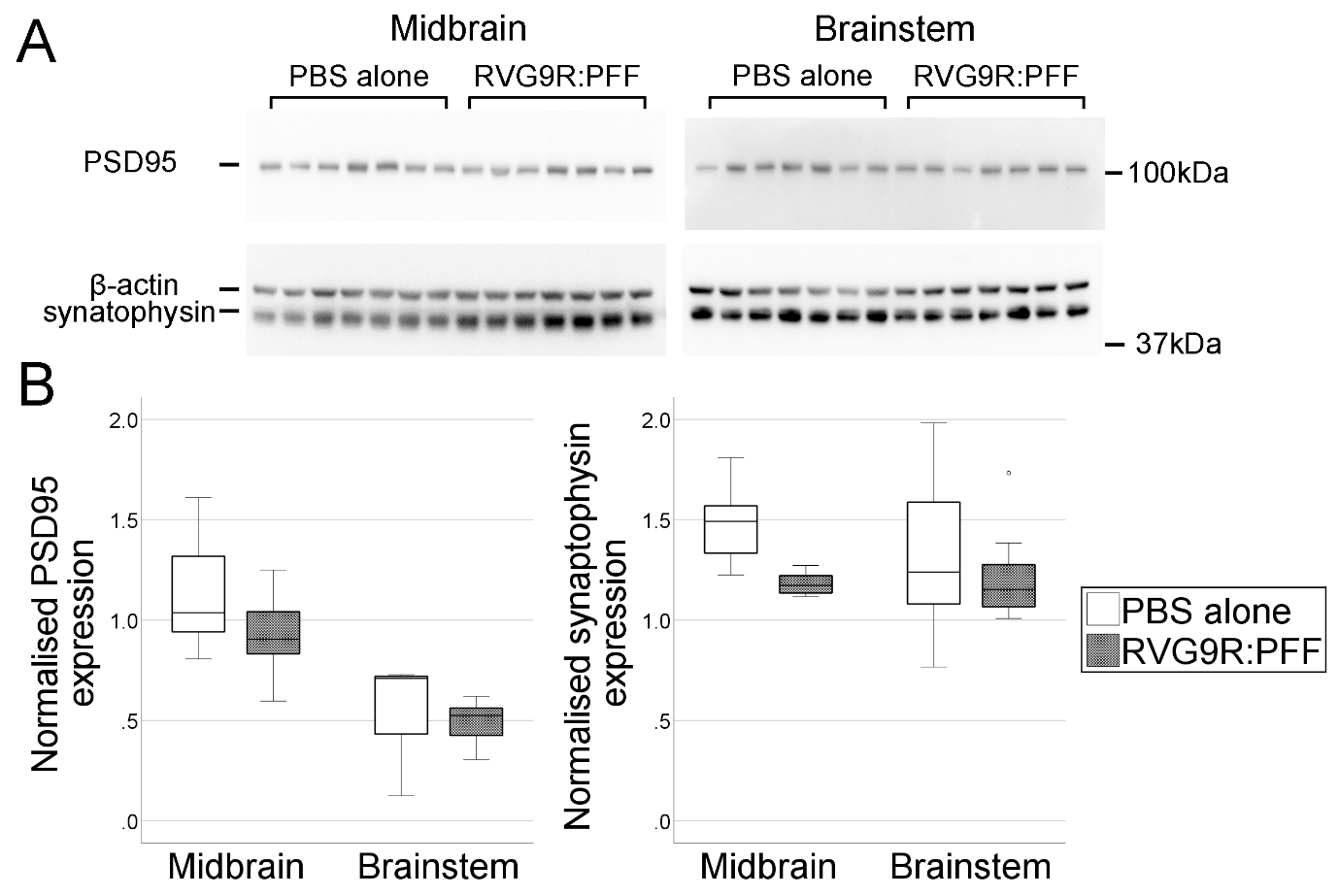


**Supplementary Figure 3.** (**A**) Representative blot images showing the expression of PSD-95 and synaptophysin, markers expressed at the post- and pre-synaptic sites, respectively, in the midbrain and brainstem of the animals. (**B**) Quantification of the Western blot results, normalised to the housekeeping β-actin protein expression, showing no significant difference in synaptic protein expression between RVG9R:PFF and control animals.


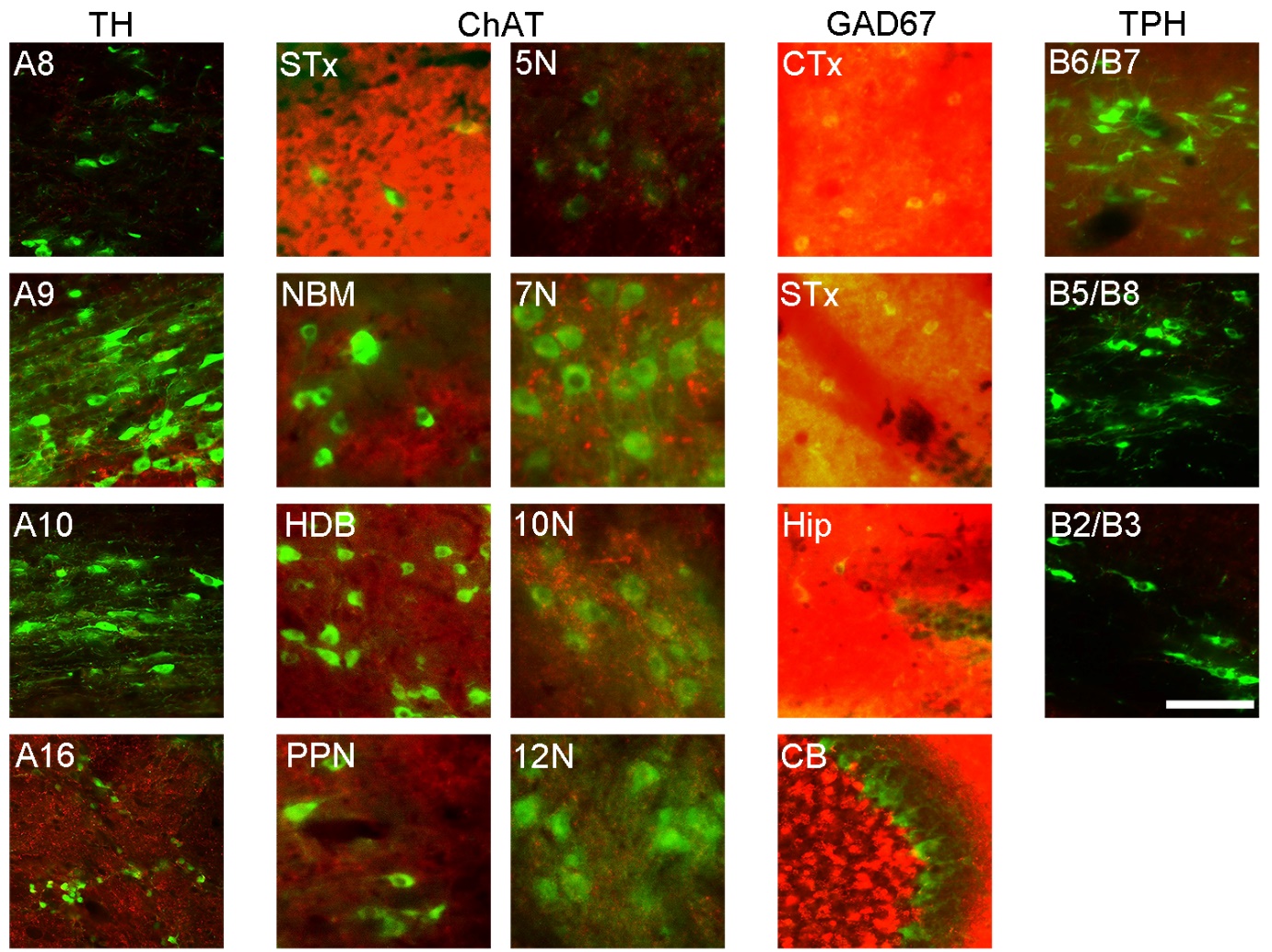


**Supplementary Figure 4.** Representative images of endogenous α-synuclein expression in individual neuronal populations. Dopaminergic, cholinergic, GABAergic, and serotonergic neurons were immunostained using TH, ChAT, GAD67, and TPH (all in green), respectively. Rodent-specific α-synuclein (D37A6) was immunostained in red, with identical imaging parameters used to individual neuronal subsets across all regions. Scale bars, 100µm.

|  |  | RVG9R:PFF | PBS alone | PFF alone |
| --- | --- | --- | --- | --- |
| Behavioural assessments | 3 months | 32 | 32 | 24 |
|  | 6 months | 32 | 32 | 24 |
|  | 9 months | 28 | 28 | 20 |
|  | 12 months | 28 | 28 | 20 |
|  | 15 months | 20 | 19 | 12 |
|  | 18 months | 20 | 18 | 11 |
| Stereological assessments | 6 months | 4 | 4 | 4 |
|  | 12 months | 8 | 8 | 8 |
|  | 18 months | 12 | 10 | 11 |

**Supplementary Table 1.** Number of animals assessed per group at individual time-points.

|  | | Sections per region | Gundersen (m=1) | | Scheaffer | |
| --- | --- | --- | --- | --- | --- | --- |
|  |  |  | Mean | S.D. | Mean | S.D. |
| Dopaminergic neurons (TH) | A8 | 4-6 | 0.120 | 0.007 | 0.145 | 0.018 |
|  | A9 | 5-6 | 0.123 | 0.053 | 0.115 | 0.033 |
|  | A10 | 5-6 | 0.085 | 0.011 | 0.094 | 0.020 |
|  | A16 | 3-5 | 0.075 | 0.022 | 0.112 | 0.044 |
| Cholinergic neurons (ChAT) | STx | 5 | 0.120 | 0.016 | 0.118 | 0.016 |
|  | NBM | 5-7 | 0.137 | 0.021 | 0.132 | 0.031 |
|  | HDB | 5-7 | 0.123 | 0.015 | 0.123 | 0.021 |
|  | PPN | 3-6 | 0.172 | 0.017 | 0.163 | 0.027 |
|  | 5N | 4-6 | 0.148 | 0.025 | 0.147 | 0.022 |
|  | 7N | 4-6 | 0.104 | 0.011 | 0.114 | 0.015 |
|  | 10N | 3-5 | 0.157 | 0.026 | 0.179 | 0.040 |
|  | 12N | 3-5 | 0.129 | 0.018 | 0.136 | 0.030 |
| GABAergic neurons (GAD67) | CTx | 5 | 0.082 | 0.012 | 0.137 | 0.025 |
|  | STx | 5 | 0.073 | 0.004 | 0.086 | 0.012 |
|  | Hip | 5 | 0.095 | 0.006 | 0.113 | 0.018 |
|  | CB | 5 | 0.088 | 0.005 | 0.144 | 0.017 |
| Serotonergic neurons (TPH) | B6/B7 | 3-4 | 0.181 | 0.031 | 0.154 | 0.020 |
|  | B5/B8 | 3-5 | 0.136 | 0.012 | 0.142 | 0.023 |
|  | B2/B3 | 3-5 | 0.125 | 0.023 | 0.164 | 0.030 |

**Supplementary Table 2.** Estimated coefficients of error values for stereological analysis of individual neuronal populations.
